# Molecular mechanisms of chemoresistance in osteosarcoma (Review)

**DOI:** 10.3892/ol.2014.1935

**Published:** 2014-03-04

**Authors:** HONGTAO HE, JIANGDONG NI, JUN HUANG

**Affiliations:** Department of Orthopedics, The Second Xiangya Hospital, Central South University, Changsha, Hunan, P.R. China

**Keywords:** osteosarcoma, chemoresistance

## Abstract

Due to the emergence of adjuvant and neoadjuvant chemotherapy, the survival rate has been greatly improved in osteosarcoma (OS) patients with localized disease. However, this survival rate has remained unchanged over the past 30 years, and the long-term survival rate for OS patients with metastatic or recurrent disease remains poor. To a certain extent, the reason behind this may be ascribed to the chemoresistance to anti-OS therapy. Chemoresistance in OS appears to be mediated by numerous mechanisms, which include decreased intracellular drug accumulation, drug inactivation, enhanced DNA repair, perturbations in signal transduction pathways, apoptosis- and autophagy-related chemoresistance, microRNA (miRNA) dysregulation and cancer stem cell (CSC)-mediated drug resistance. In addition, methods employed to circumvent these resistance mechanism have been shown to be effective in the treatment of OS. However, almost all the current studies on the mechanisms of chemoresistance in OS are in their infancy. Further studies are required to focus on the following aspects: i) Improving the delivery of efficacy through novel delivery patterns; ii) improving the understanding of the signal transduction pathways that regulate the proliferation and growth of OS cells; iii) elucidating the signaling pathways of autophagy and its association with apoptosis in OS cells; iv) utilizing high-throughput miRNA expression analysis to identify miRNAs associated with chemoresistance in OS; and v) identifying the role that CSCs play in tumor metastasis and in-depth study of the mechanism of chemoresistance in the CSCs of OS.

## 1. Introduction

Osteosarcoma (OS) is the most common malignant primary bone tumor in children and adolescents. OS has a predilection for the metaphyseal portions of the long bone, with the distal femur and proximal tibia accounting for ~50% of all cases (1). OS is highly aggressive and it metastasizes primarily to the lung (2). In ~75% of cases, patients with OS are between 15–25 years old. The median age of an OS patient is 16 years old, with a male predominance. The high incidence of OS during the adolescence growth spurt indicates there may be a link between this disease and bone development. The incidence of OS is also increased in patients with germline mutations in the retinoblastoma and P53 genes, indicating that these genes may be involved in the occurrence of the disease. Histologically, OS is characterized by a proliferation of malignant spindle cells. Although several histological subtypes may exist, including chondroblastic and fibroblastic OS, once the osteoid directly produced by sarcoma cells is found, OS can be diagnosed (3). With regard to the clinical features, pain and swelling of the soft tissue are the most common symptoms of OS patients. Up to 20–25% of recently diagnosed patients present with metastases detectable by radiography, which mainly occur in the lung. Prior to the use of adjuvant and neoadjuvant chemotherapy, the long-term survival rate subsequent to surgical resection alone was <20%. Luckily, multi-agent chemotherapy regimens that were pioneered in the 1970s and early 1980s have dramatically improved the survival rate by up to ~70%, and the necessity for the chemotherapy used for OS patients has been demonstrated by randomized controlled trials (4). The current national and international co-operative trial for patients with recently diagnosed OS mainly builds upon the backbone of cisplatin, doxorubicin and methotrexate (MTX) treatment. Due to the combination of these anti-OS drugs, the 5-year survival rate in patients with localized disease is ~70%. However, the survival rate has plateaued since the mid-1980s, despite advances in anti-OS therapy. In addition, patients with metastatic or recurrent diseases have a <20% chance of long-term survival despite aggressive therapies. These figures have changed little in the past 30 years (5). To a certain extent, the reason behind this may be ascribed to the chemoresistance to anti-OS therapy. The development of chemoresistance in malignant tumors limits the effectiveness of cytotoxic drugs, and this is particularly true in OS, which is characterized by the frequent refractoriness to chemotherapy. Therefore, elucidation of the mechanisms of chemoresistance and implementation of strategies to overcome chemoresistance will definitely play a pivotal role in improving the survival rate of OS patients. This review mainly focuses on the recent studies on the mechanisms of chemoresistance in OS and the methods to overcome chemoresistance.

## 2. Decreased intracellular drug accumulation

**Figure 1:**
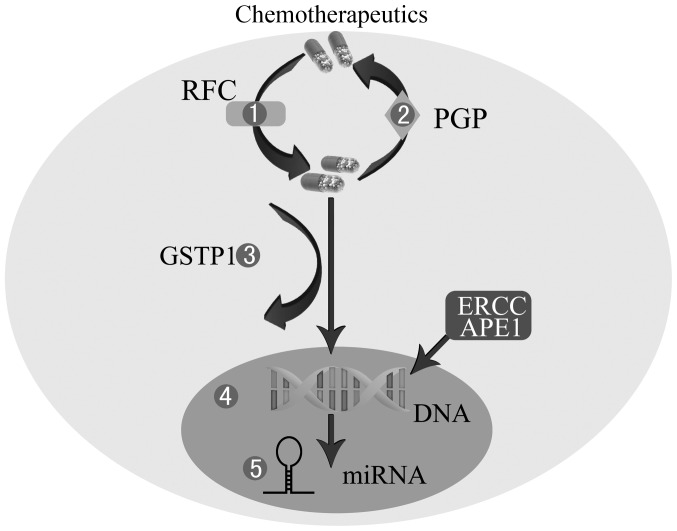
Mechanisms of chemoresistance in OS. 1, Decreased intracellular drug accumulation mediated by lower RFC. 2, Increased efflux of drugs through P-GP. 3, Drug inactivation by GSTP1. 4, Enhanced DNA repair by APE1 or ERCC. 5, miRNA dysregulation. OS, osteosarcoma; RFC, reduced folate carrier; P-GP, P-glycoprotein; GSTP1, glutathione S-transferase P1; APE1, apurinic endonuclease 1; ERCC, excision repair cross-complementing; miRNA, microRNAs.

The mechanism behind how the majority of chemotherapy drugs are absorbed by the tumor cells is unclear. Impaired transport of MTX, an effective inhibitor of dihydrofolate reductase, is a common mechanism of resistance in OS (6). As MTX enters cells through the reduced folate carrier (RFC) at the cell membrane, the decreased expression of RFC is proven to be associated with MTX-resistance (Fig. 1) (7). In one study, decreased RFC expression was found in 65% of OS biopsy samples, and decreased RFC expression was more commonly found in samples with a poor histological response to chemotherapy (8). Another study demonstrated that RFC1 expression was moderately decreased in OS samples with a poor histological response to pre-operative treatment, and RFC expression was also lower in initial OS biopsy specimens compared with metastatic specimens (9). A subsequent confirmatory study assessing the RFC protein level found that the protein levels of RFC were lower in primary OS biopsy samples than recurrent tumor specimens, and tumors with poor histological responses to pre-operative chemotherapy exhibited significantly lower RFC levels at diagnosis than those with favorable responses. However, notably, post-chemotherapy progression to recurrence was associated with a significant increase in RFC expression (10). Subsequently, the functionality of the altered RFC proteins has been studied. One of the altered RFC proteins, Leu291Pro, has been demonstrated to confer drug resistance since the carrier is unable to translocate the substrate across the cell membrane, and three alterations, Ser46Asn, Ser4Pro and Gly259Trp, confer a certain degree of resistance to MTX via a decreased rate of transport (11). Furthermore, studies that have focused on the RFC gene have also been reported. Analysis of the RFC gene copy number by dot blot and Southern blot has not identified any variation between the parental cell lines and their MTX-resistant variants, indicating that the reduced RFC expression was not due to gene deletion (12). Sequence alterations in the RFC have been observed, and OS tumor samples with RFC sequence alterations have been shown to possess significantly higher frequencies of inferior histological response (Huvos grade I or II). However, in a study by Yang *et al* (13), it was not clear if any of these sequence alterations were germline- or tumor-specific, as normal tissue and peripheral blood were not obtained. Although the controversy about RFC remains, trimetrexate, a novel antifolate that does not require the RFC for transport into cells, was enrolled in a phase II study of relapsed or refractory OS patients, and was demonstrated to be effective in 5 out of 38 (13%) patients. In addition, a phase I trial of a combination of trimetrexate and high-dose MTX in patients with recurrent OS is ongoing (14).

Another mechanism leading to decreased intracellular drug accumulation in numerous tumors is the non-specific removal of cytotoxic drugs from tumor cells by the membrane pump P-glycoprotein (P-GP) (15). This membrane-associated protein, a high molecular weight protein of 170 kD coded by the multidrug-resistant (MDR) human gene known as MDR1, belongs to the ATP-binding cassette (ABC) transporters, and is considered to act as an efflux pump extruding drugs from the cell (Fig. 1) (16). A series of studies has found that the high expression of P-GP may be responsible for doxorubicin resistance in human or canine OS cell lines (17–19). Additionally, several retrospective studies have indicated that the overexpression of P-GP appeared to be associated with tumor progression, a higher relapse rate and a trend towards a worse outcome (20,21). By contrast, other studies have found no correlation between P-GP expression and tumor progression or event-free survival (22,23). Similarly, a prospective, multicenter study of 123 non-metastatic OS patients did not reveal any correlation between P-GP mRNA expression and the risk of disease progression or relapse (24). A meta-analysis conclusively showed that P-GP was not associated with the histological responses of OS patients treated with a combination of chemotherapy regimens (25). Subsequently, a comparative clinical pathological study examined histological biopsies from 117 patients and found that P-GP expression could not serve as a predictor of treatment response or survival rate of OS patients (26). Furthermore, in OS cell lines transfected with the MDR gene, an association has been shown between the increased expression of P-GP and a low aggressive phenotype (27). In order to overcome the resistance mechanism caused by P-GP, recent studies have focused on a novel drug delivery system, consisting of a biocompatible and lipid-modified polymeric nanoparticle. The initial results have indicated that this nanoparticle is a promising platform for delivering doxorubicin and small interfering RNAs (siRNAs) to the drug-resistant OS cell lines, which may reverse the decreased intracellular drug accumulation mediated by P-GP (28–30).

## 3. Drug inactivation

Human glutathione S-transferase P1 (GSTP1), one of the cytosolic GSTs that belong to a major group of the phase II detoxification enzyme superfamilies, inactivates a variety of exogenous xenobiotics, including mutagens, anticancer agents and their metabolites (Fig. 1) (31). It is believed that the overexpression of GSTP1 is linked to chemoresistance in numerous cancers (32). A study found that OS-bearing dogs with higher GSTP1 expression had significantly shorter median remission and survival times than dogs with a lower expression of GSTP1 (33). In another study of human OS specimens obtained from 60 OS patient cases, an association was shown between the overexpression of GSTP1 at surgery and a poor histological response to pre-operative chemotherapy (34). Similarly, another study also found that chemotherapy can induce the upregulation of GSTP1 protein expression, and that the high expression of GSTP1 is associated with a poor prognosis (35). In addition, the mRNA expression levels of GSTP1 in human OS xenografts have been assessed, and a significant correlation was shown between a higher GSTP1 expression and a low growth inhibition of OS cells treated with doxorubicin (36). Furthermore, a study by Huang *et al* (37) indicated that the protective role of GSTP1 in OS cell survival may be mediated in part by promoting the activation of extracellular signal-regulated kinase (ERK)1/2 rather than c-Jun N-terminal kinases (JNK) in HOS OS cells triggered by doxorubicin or cisplatin. Windsor *et al* (38) investigated the association of 36 candidate genetic polymorphisms in MTX, adriamycin and cisplatin pathway genes with the histological response and survival rate in OS patients and found that a poor histological response was increased in variants of GSTP1, c.313A>G p.lle(105)Val. A study by Zhang *et al* (39) also showed that individuals with the GSTP1 Val/Val genotype tended to live for less time than those with the IIe/IIe genotype. However, a study by Yang *et al* (40) found that the GSTP1 Val genotypes exhibited significantly higher rates of response to chemotherapy. These results indicate that GSTP1 polymorphisms may be candidate pharmacogenomic factors to be explored in the future to benefit OS patients with chemotherapy.

To overcome GSTP1-related resistance in OS, the *in vitro* effectiveness of 6-(7-nitro-2,1,3-benzoxadiazol-4-ylthio)hexanol (NBDHEX), a highly efficient inhibitor of GSTP1, was tested. A study found that NBDHEX was extremely active in the resistance to doxorubicin and MTX in the U-2OS or Saos-2 cell lines (41). A further study on NBDHEX confirmed that the *in vitro* activity of NBDHEX was mostly associated with cytostatic effects, with less evident apoptotic induction and a positive effect against the metastasization of OS cells (42). Subsequently, a proteomic investigation was performed and the result demonstrated that NBDHEX was able to dissociate the GSTP1-tumor necrosis factor receptor-associated factor (TRAF)2 complex, which restores the TRAF2/apoptosis signal-regulating kinase 1 (Arabidopsis) signaling, thereby leading to the simultaneous and prolonged activation of JNK and p38 (43). These findings may support the fact that targeting GSTP1 with NBDHEX may be a promising novel therapeutic possibility for OS patients.

## 4. Enhanced DNA repair

Chemotherapeutic agents routinely used in the therapy of OS, including cisplatin and cyclophosphamide, work by damaging DNA. Therefore, one of the mechanisms associated with the resistance to these drugs is the enhanced capacity of the cell to carry out repair on damaged DNA (Fig. 1). In general, cells repair DNA damage via four main mechanisms: Direct reversal, base excision repair, nucleotide excision repair and mismatch repair.

Apurinic endonuclease 1 (APE1), one of the main enzymes involved in the base excision repair pathway, has been linked to chemosensitivity and prognosis in a number of cancers, including glioma, melanoma and cervical, prostate and bladder cancer (44–47). High expression levels of APE1 have been demonstrated to significantly correlate with the reduced survival times of OS patients, and decreased APE1 levels in siRNA-treated human OS cells have led to enhanced cell sensitization to the DNA damaging agents (48). Similarly, decreased APE1 levels mediated by siRNA also enhance the sensitivity of human OS cells to endostatin *in vivo* (49). Subsequent to these findings, a study found that the APE1 gene is amplified in siRNA and APE1 expression, and can serve as an independent predictor of OS patients with local recurrence or metastasis (50). Furthermore, to overcome the increased resilience in cells to chemotherapy caused by APE1, small molecule inhibitors of the APE1 endonuclease, including lucanthone, 7-nitroindole-2-carboxylic acid, resveratrol and arylstibonic acids, have been gradually reported (47,51–53). However, these inhibitors are either fairly weak or non-specific, or the effects in cell culture are difficult to reproduce (54). Therefore, the development of effective small molecule inhibitors of APE1 may benefit OS patients.

The excision repair cross-complementing (ERCC) set of proteins, including ERCC1, 2 and 4, belongs to the nucleotide excision repair system. A study has shown a correlation between ERCC4 and the histological response to chemotherapy in OS patients (55). Similarly, the expression of ERCC4 and ERCC2 mRNA in OS cells has been shown to correlate with the chemotherapeutic effect in OS patients (56). Subsequent to these findings, a study found that a polymorphism in the ERCC2 gene was associated with a positive tumor response and survival rate in cisplatin-treated OS patients (57). Another study of common polymorphisms also found a positive association between ERCC2 polymorphisms and an increased event-free survival rate, and the result indicated that the variant allele of ERCC2, rs1799793, could serve as a marker of OS associated with an improved prognosis following platinum therapy (58). In addition, an association between polymorphisms in ERCC2 and an improved cisplatin response and survival rate in OS patients was also shown in a Chinese population (59).

## 5. Perturbations in signal transduction pathways

**Figure 2:**
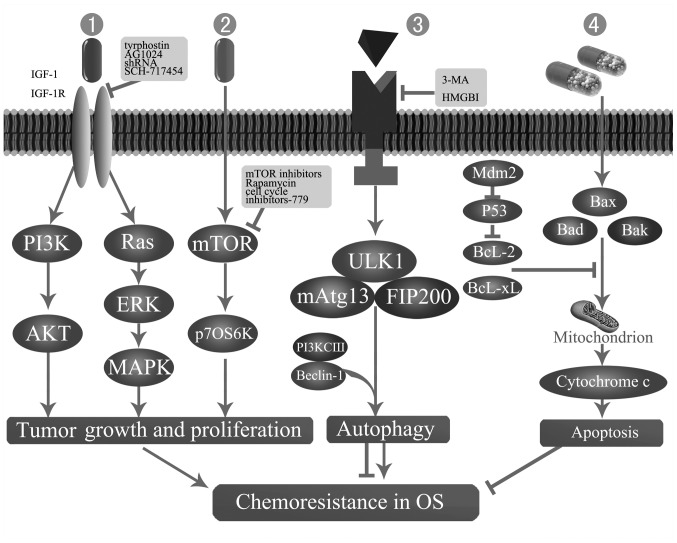
Mechanisms of chemoresistance in OS. 1 and 2, Perturbations in mTOR or IGF-IR signal transduction pathways. 3 and 4, Apoptosis and autophagy-related chemoresistance. 3-MA, 3-methyladenine; AKT (PKB), protein kinase B; Bad, basal cell lymphoma 2-associated death protein; Bak, basal cell lymphoma 2 homologous antagonist killer protein; Bax, basal cell lymphoma 2-associated X protein; Bcl-2, basal cell lymphoma 2 protein; Bcl-xl, basal cell lymphoma extra large protein; ERK, extracellular signal-regulated kinase; FIP200, family interacting protein of 200 kDa; HMGB1, high mobility group box 1 protein; IGF-I, insulin-like growth factor I; IGF-IR, IGF-I receptor; MAPK, mitogen activated protein kinase; mAtg13, mammalian autophagy-related gene 13; Mdm2, murine double minute 2; mTOR, mammalian target of rapamycin; OS, osteosarcoma; p70S6K, ribosomal protein S6 kinases, 70 kDa; PI3K, phosphoinositide 3-kinase; PI3KCIII, PI3K class III; ULK1, Unc-51-like kinase 1.

Perturbations in signal transduction pathways are likely to be involved in the chemoresistance of tumors. One pathway that has been intensely studied is the mammalian target of rapamycin (mTOR) pathway (Fig. 2). The serine-threonine kinase, mTOR, plays a major role in the regulation of protein translation, cell growth and metabolism. Alterations of the mTOR signaling pathway are common in various cancers, including OS, and the mTOR signaling pathway is being actively pursued as a therapeutic target (60). In OS cells lines from dogs, mTOR and its downstream product have been shown to be present and active, and pathway inhibition by rapamycin decreased the survival rate of the tumor cells (61). In the human OS cell lines, HOS and KHOS/NP, the mTOR inhibitor, rapamycin, downregulated the activity of mTOR and strongly inhibited cell growth, as apparent by an increase in the G_1_ phase and a decrease in the S-phase of the cell cycle, linked with the downregulation of cyclin D1 (62). Clinical studies have also been started. A correlation has been shown between the mTOR/p70S6K signal transduction pathway and OS patient prognosis, indicating the prognostic significance of the mTOR/p70S6K signal transduction pathway (63). In an initial testing (phase I) of rapamycin by the pediatric pre-clinical testing program (PPTP), rapamycin was demonstrated to possess broad anti-tumor activity against the PPTP tumor panels *in vivo,* including that of OS (64). In a murine model of OS, the blockade of the mTOR pathway with rapamycin or its analog, cell cycle inhibitor-779, led to a significant inhibition of experimental lung metastasis *in vivo* (65). In addition, a recent study has revealed that rapamycin treatment reduces the gene expression of vascular endothelial growth factor (VEGF) and bone morphogenetic protein-2, and that it inhibits the invasion, proliferation and migration of murine K7M2 OS cells *in vitro* (66). These results indicate that the mTOR pathway may not only decrease the survival of OS tumor cells, but that it also plays a significant role in metastasis.

The insulin-like growth factor I receptor (IGF-IR), a transmembrane receptor with tyrosine kinase activity, is involved in the initiation and progression of a variety of cancers (67). Activation of the phosphorylation of IGF-IR leads to subsequent activation of at least two pro-survival signaling pathways. Following IGF-1R phosphorylation, stimulation of the phosphoinositol 3-kinase (PI3K)-protein kinase B signaling pathway is the main event, which leads to cell survival. The second pathway consists of Ras, Raf and ERK/mitogen-activated protein kinase (MAPK) activation, which leads to proliferation and tumor growth (Fig. 2) (68). These two key downstream pathways of the IGF-IR have also been demonstrated to be activated in OS cell lines (69). Pre-clinical data has indicated that IGF-IR may constitute a significant therapeutic target in a variety of pediatric solid tumors, including neuroblastoma and musculoskeletal tumors cells (70,71). Similarly, IGF-1R has been found to be abundantly expressed in OS, indicating that the inhibition of IGF-IR may be effective in the therapy of OS (72). A study by Luk *et al* (73) indicated that IGF-1R inhibition by tyrphostin AG1024 together with doxorubicin achieves an additive anti-OS growth effect, accompanied with increased apoptosis, cytotoxicity and dual cell cycle arrest, which indicates that IGF-1R inhibition can enhance the effect of doxorubicin chemotherapy in OS cell lines. Another study by Wang *et al* (74) has shown that targeting IGF-1R using lentivirus-mediated short hairpin RNA could lead to growth suppression and the enhanced caspase-3-mediated apoptosis of OS cells not only *in vitro*, but also *in vivo*. In addition, a recent study indicated that IGF-1R was involved in the *in vitro* behavior of metastatic OS cell lines (75). A subsequent study found that the expression of the IGF-1R protein was closely associated with the surgical stage and distant metastasis of OS patients, and that IGF-1R can be used as an independent prognostic marker for OS patients (76).

Although the resistance mechanism of IGF-1R inhibitors remains largely unclear, candidate drugs, including monoclonal antibodies, small molecule tyrosine kinase inhibitors and ligand binding antibodies, are being introduced in phase I and II studies for a wide variety of cancers (77). Ewing sarcoma provides the most clear evidence of clinical activity. The results of a recently published phase II trial found that AMG 479 (a fully human monoclonal antibody to IGF-1R) achieved a clinical benefit rate of 17% in recurrent refractory Ewing’s family tumors (78). In addition, the efficacy of SCH-717454 (robatumumab, a fully human neutralizing anti-IGF-1R antibody) in OS patients is planned to be investigated in a phase II trial, and the result of the study is eagerly awaited (79).

Additionally, other receptor tyrosine kinases, including human epidermal growth factor receptor 2 (HER2/neu) and VEGF have also been recognized as potential targets for the therapy of OS, as studies have shown that HER2/neu and VEGF expression correlate with the malignant phenotype in OS (80,81). Cediranib (AZD-2171), a specific VEGF receptor inhibitor, has been demonstrated to possess a growth inhibitory effect in solid tumor xenograft models, including those of OS (82). However, in a phase II trial of the HER2-targeted agent trastuzumab in combination with cytotoxic chemotherapy for treatment of metastatic OS patients, no significant difference was found between the HER2-positive and HER2-negative groups (83). Therefore, the therapeutic benefit of this HER2-targeted agent remains uncertain, and a definitive assessment of the potential role of trastuzumab in treating OS requires further studies of patients with HER2-positive disease.

## 6. Apoptosis and cell cycle-related gene expression turbulence

Apoptosis is the primary mode of cell death induced by chemotherapy. Conversely, cell cycle arrest allows the host cell to repair its damaged DNA prior to cell division, while cells with excessive DNA damage undergo apoptosis. Therefore, cell cycle and apoptosis-related gene expression may be involved in the modulation of chemotherapeutic cytotoxicity (Fig. 2).

The P53 gene, which plays a pivotal role in cell cycle arrest and in the regulation of apoptosis has been demonstrated to be involved in the modulation of anticancer drug cytotoxicity (84). Wild-type or mutant P53 genes were shown to be associated with the chemoresistance in OS cells (85). However, whether the P53 gene takes part in the elevated or decreased chemoresistance in OS has been confusing. A Study by Wong *et al* (86) showed that the transfection of a mutated form of P53, p53-R273H, can downregulate the procaspase-3 level and induce resistance to drug toxicity in the p53-null human Saos-2 cell line. However, the various available studies have not yielded consistent results. A study by Fan and Bertino revealed that the induction of p53 conferred resistance to cisplatin when OS cells were cultured in media containing normal serum concentrations, whereas p53 induction led to increased cisplatin sensitivity when cells were grown in low serum media (87). A study by Tsuchiya *et al* (88) demonstrated that the human OS cell line, Saos2, transfected with wild-type p53, was twice as sensitive to cisplatin as the parental cells. Furthermore, another study revealed that the enhanced expression of murine double minute 2 (Mdm2), a downstream mediator of p53, may inhibit p53-mediated apoptosis and endow cells with resistance to DNA damaging agents (89). A further study found that modified p53, particularly p53 14/19, retains the pro-apoptotic and transcriptional activity of wide-type p53, and augments the effectiveness of chemotherapy even in cells overexpressing Mdm2 (90).

Contradictory results also exist between studies that focus on the expression of P53 in clinical OS patients. OS patients with a p53 gene deletion were found to be more sensitive to pre-operative chemotherapy compared to those without such gene loss (91). Similarly, several studies demonstrated a direct correlation between p53-positive expression and the resistance to therapy or the survival of OS patients, and concluded that p53 expression may be a useful prognostic factor in OS patients (92,93). However, a prospective study found no evidence that P53 mutations can predict the development of metastases, chemotherapy response and clinical outcome in patients with high-grade OS (94). Therefore, additional studies are required to obtain an improved explanation.

B-cell lymphoma 2 (Bcl-2) is the founding member of a family of proteins associated with cell death signaling, and was first isolated as the product of an oncogene (95). The Bcl-2 protein family is comprised of anti-apoptotic proteins, including Bcl-2 and Bcl-xL, and pro-apoptotic proteins, including Bax, Bak and Bad (96). These proteins mainly regulate apoptosis at the mitochondrial outer membrane and control the initiation of mitochondrial outer membrane permeabilization (97). Studies have shown that Bcl-2 and Bax have a role in affecting drug-induced apoptosis and regulating the resistance to chemotherapy in various tumor cells, including hepatocellular carcinoma and bladder, lung and ovarian cancer (98–101).

*In vitro* studies of anti-apoptotic proteins, the downregulation of Bcl-2 and Bcl-xL by lentivirus-mediated Bcl-2-knockdown or stable transfection with Bcl-xL cDNA could significantly enhance the *in vitro* chemosensitivity of OS cells to doxorubicin and cisplatin (93,102,103). A synergistic effect, created by co-silencing Bcl-2 and cyclin D1, was also found to enhance the chemosensitivity of OS cells (104). Subsequently, a study revealed that Bcl-xL may exert an anti-apoptotic effect by stimulating oxidative phosphorylation or inhibiting caspase activation (105). Conversely, downregulation of a pro-apoptotic protein, Bax, by lentivirus-mediated knockdown decreases the *in vitro* chemosensitivity of OS cells (106,107). In addition, upregulated Bax gene expression by runt-related transcription factor 2 (Runx2), which can directly bind to two Runx-specific regulatory elements on the human Bax promoter, could increase the apoptosis and drug sensitivity of OS cells (108). Additionally, another study has demonstrated that although Bax expression is not affected by the knockdown of c-Myc or caspase-2, since caspase-2 is important for cytosolic Bax to integrate into the outer mitochondrial membrane, and as c-Myc is critical for the oligomerization of Bax, that during cytotoxic drug-induced apoptosis, c-Myc and caspase-2 remain involved in activating Bax (109).

In clinical trials, a higher cellular expression level of Bcl-2 has been shown in high-grade OS patients with recurrent pulmonary metastases compared with those with primary tumors, and the expression of Bcl-2 was also shown to be closely associated with the prognosis of OS patients (110,111). Subsequently, a higher mRNA expression level of Bcl-xL was found to significantly correlate with an advanced clinical stage and a poorer survival rate in OS patients (112). However, although Bc1–2 is highly expressed in the specimens of OS patients, no correlation between the expression of Bc1–2 and chemosensitivity and the overall survival of high-grade OS patients was shown in the study by Nedelcu *et al* (113). Similarly, Bax and Bcl-2 protein expression was observed in OS patients, but proteins were found to be unable to predict the overall or disease-free survival rate. Nevertheless, an increased Bax/Bcl-2 protein expression ratio was associated with a decreased 4-year survival and disease-free survival rate of OS patients (114,115).

## 7. Autophagy-related chemoresistance

Autophagy is a homeostatic and evolutionarily conserved process that degrades cellular organelles and proteins and maintains cellular biosynthesis (116). This process can be triggered under physiological conditions, including nutrient starvation and growth factor deprivation, or in response to a variety of stress stimuli, including hypoxia and the exposition to cytotoxic compounds (117). Autophagy has been referred to as a double-edged sword. On one hand, it allows tumor cells to survive bioenergetic stress via clearance of damaged organelles and proteins under adverse conditions (116). On the other hand, in certain cellular contexts, sustained or excessive tumor cell autophagy promotes programmed cell death, particularly in apoptosis-defective cells, although certain studies have considered autophagic cell death to be a misnomer (118,119). In recent years, numerous studies have focused on the association between autophagy and the chemoresistance of tumor cells. In leukemia and colon cancer cell lines, the inhibition of autophagy was shown to sensitize resistant cells to tumor necrosis factor-related apoptosis-inducing ligand-mediated apoptosis (120). In addition, the ability of autophagy inhibition to enhance chemosensitivity and tumor regression was confirmed in various animal models. Firstly, the inhibition of autophagy by chloroquine was shown to increase apoptosis and enhance tumor cell death in lymphoma, colon cancer and prostate cancer xenograft mouse models (121–123). Secondly, autophagy inhibition triggered by 3-methyladenine (3-MA) increased apoptotic induction by 5-fluorouracil in association with tumor regression in colon cancer xenografts (124). Thirdly, multiple studies have revealed that the inhibition of autophagy by the knockdown of autophagy-related genes can effectively enhance tumor cell death induced by diverse anti-cancer drugs in pre-clinical models (125,126).

Subsequent studies on the dual role of autophagy in OS have been published. A study by Lambert *et al* (128) found that induction of autophagy was shown in U2OS cells treated with doxorubicin and roscovitine, and it was considered that autophagy may be the cause of increased cytotoxicity. One study by Meschini *et al* (129) demonstrated that autophagy induced by a natural product, bisindolic alkaloid voacamine, showed a lethal effect, which is effective against drug-resistant OS cell lines either used alone or in association with conventional chemotherapeutics. A study by Kim *et al* (130) found that in the Saos-2 cell line, the inhibition of autophagy along with 3-MA significantly increased paclitaxel (PCX)-induced apoptotic cell death. It was indicated that a combination of treatment involving autophagy inhibitor therapy and low-dose PCX therapy could be an effective and potent strategy for improving the chemotherapy for OS, although PCX has not been incorporated in the current protocols for OS treatment. By contrast, a study by Zhang *et al* (131) found that following the downregulation of autophagy in the MG63 cells by the autophagy inhibitor, 3-MA, the chemotherapeutic sensitivity of MG63 cells treated with cisplatin was enhanced, which indicated that autophagy may have a protective effect on OS cells. Similarly, a study by Coupienne *et al* (132) found that autophagy protected OS cells against photodynamic therapy-induced cell death and thus provided an improved survival rate for the OS cells. The protective role of OS cells mediated by autophagy was also shown in studies from the Central Laboratory of the Second Xiangya Hospital (Changsha, China). The results demonstrated that autophagy induced by the high mobility group box 1 protein (HMGB1), a highly conserved nuclear protein, increased chemoresistance to conventional anti-OS agents, including doxorubicin, cisplatin and MTX. Subsequently, our further studies identified that HMGB1 bound to the autophagy regulator Beclin1, and the interaction between HMGB1 and Beclin1 was dependent upon the autophagic complex, ULK1-mAtg13-FIP200. The formation of the Beclin1-PI3K class 3 complex that facilitates autophagic progression was also regulated by HMGB1 (Fig. 2) (133,134).

## 8. microRNA (miRNA) dysregulation

miRNAs are a class of small non-coding regulatory RNA molecule that have recently been shown to be involved in a wide array of biological processes (135). The abnormal expression of miRNA has been indicated to be associated with various cancers (136,137). When miR-34a expression was enforced, as shown by the functional analysis of miR-34a in Ewing’s sarcoma cell lines, this indicated that the cells were sensitized to doxorubicin and vincristine (138). A study by Gougelet *et al* (139) found that OS of rat and human origins showed an miRNA signature that could discriminate promising from unpromising responders for ifosfamide treatment. The study also identified five discriminating miRNAs (miR-92a, miR-99b, miR-132, miR-193a-5p and miR-422a) in tumors of OS patients, which could be used as a potent diagnostic tool to predict tumor sensitivity to ifosfamide. In addition, a study by Song *et al* (140) found that the expression of miR-140 was involved in the chemoresistance to OS xenografts by reduced cell proliferation via G_1_- and G_2_-phase arrest. Their subsequent study indicated that G_2_ arrest was induced by a decrease in cell proliferation stimulated by miR-215 via the suppression of denticleless protein homolog expression, which resulted in an increase in MTX-chemoresistance in the human OS cell lines, U-2 and MG63 (141). Furthermore, a study by Cai *et al* (142) found that miR-15a and miR-16-1 downregulated cyclin D1 and induced apoptosis and cell cycle arrest in OS, indicating that miR-15a and miR-16-1 may be used for OS therapy (Fig. 1).

## 9. Cancer stem cells (CSCs) and drug resistance

The CSC hypothesis, first proposed ~50 years ago, postulates that a small subpopulation of cancer cells with an unlimited proliferative capacity drives tumor self-renewal and differentiation (143). However, no substantial progress was made in the CSC hypothesis until Bonnet and Dick (144) first isolated a subpopulation of human acute myeloid leukemia cells with a CD34^++^/CD38^−^ phenotype, where CD34^++^ has a stronger affinity to the antigen. Subsequently, CSCs have been shown to be indicated in the pathogenesis of numerous tumors, including leukemia, brain tumors and cortical glial tumors (144–146). Studies have also found that CSCs may be involved in the mechanisms of chemoresistance (147–149).

Although the specific role that CSCs play in the chemoresistance of OS cells has not been clearly elucidated, several of the aforementioned mechanisms could mediate the intrinsic chemoresistance in CSCs. A study by Di Fiore *et al* (150) found that a novel CSC cell line, 3AB-OS, irreversibly selected from human OS MG-63 cells by long-term treatment with 3-aminobenzamide (3AB), expressed higher levels of the ABC transporter, ABCG2 (a drug resistance marker), with a high-drug efflux capacity and anti-apoptosis genes, including FADD-like apoptosis regulating protein-L, Bcl-2, X-linked inhibitor of apoptosis protein, inhibitor of apoptosis proteins and survivin. A study by Fujii *et al* (151) found that the MG63 OS cell line possessed an ability to form clonal expanding colonies (sarcospheres), which show a strong resistance to doxorubicin and cisplatin due to the increased expression of the DNA repair enzyme genes, MutL homolog 1 and MutS protein homolog 2. Additionally, caffeine, a DNA repair inhibitor, enhanced the efficacy of these drugs, indicating that the drug resistance in sarcosphere cells was partly associated with the efficient DNA repair ability. Their subsequent study indicated that CSCs and the sarcosphere cells from the MG63 cell line showed a strong chemoresistance against doxorubicin and cisplatin, which may be attributed to the efficient detoxification by elevated aldehyde dehydrogenase 1 mRNA expression (152). In addition, a study by Martins-Neves *et al* (153) showed that OS cells contained a CSC population relatively resistant to doxorubicin and MTX, and this resistant phenotype appeared to be associated with the high expression of the drug efflux transporter, P-GP.

## 10. Conclusions

Although great progress has been made by combination chemotherapy and aggressive surgical resection in the treatment of OS, the survival rate of OS patients with localized disease at diagnosis has plateaued at ~70% since the mid-1980s, and the long-term survival rate of patients with metastatic or recurrent disease remains at <20% (154). Accordingly, an improved understanding of the molecular mechanisms of chemoresistance and the identification of novel strategies to circumvent the resistance mechanisms are desperately required. As mentioned in the present review, chemoresistance in OS has been shown to occur by a variety of mechanisms, including decreased intracellular drug accumulation mediated by RFC or P-GP, drug inactivation by GSTP1, enhanced DNA repair by APE1 or ERCC, perturbations in mTOR or IGF-IR signal transduction pathways, apoptosis and autophagy-related chemoresistance, miRNA dysregulation and CSC-mediated drug resistance. In addition, the interaction between OS cells and their micro-environment has also been shown to be involved in the chemoresistance in OS, and therapies disrupting this interaction have been demonstrated to be efficacious in OS treatment in pre-clinical studies. However, almost all these studies on the mechanisms of chemoresistance in OS are at an early stage, and further studies are eagerly anticipated on the following aspects.

On the basis of the current understanding of the mechanism of resistance mediated by RFC, a novel antifolate, trimetrexate, which does not require the RFC for transport into cells, has been already demonstrated to be effective in OS patients in a phase II study (14). Prior to its use in clinical trials, further clinical studies are required to assess the effect of trimetrexate in OS patients either used alone or in combination with other anti-OS drugs. To circumvent the mechanism of resistance mediated by P-GP and to improve intracellular drug accumulation, novel delivery patterns, including biocompatible nanoparticles and liposomal encapsulation, have emerged and have been shown to improve delivery efficacy in several studies (155). Further studies should be focused on the co-administration of nanoparticles combined with conventional chemotherapy and an efflux pump inhibitor, and the precise mechanism of the interaction between these drugs also deserves further investigation.

In the past two decades, studies about the signal transduction pathways and targets involved in the malignant behavior of OS led to the development of a variety of novel targeted therapeutic agents for OS, including IGF-1R antibodies and mTOR inhibitors (65,70). The following challenge is to identify promising agents in the treatment of OS, which requires more trials for successful design and completion. In addition, an improved understanding of the targeted molecules of signal transduction pathways that regulate cell proliferation and growth and the interaction between these pathways will lead to the development of numerous novel targeted agents.

The association between autophagy and chemoresistance in tumors attracts more and more attention in studies. However, the exact role that autophagy plays in cancer drug-resistance remains controversial, and studies on the autophagy and chemoresistance of OS remain rare (156). Similarly, little is known about the autophagy-related pathways and the association with apoptosis. Therefore, elucidating the signaling pathways of autophagy and the association with apoptosis in OS cells is definitely of great significance, and will bring a novel perspective on the therapy of OS.

Recently, miRNA has become a hot spot in the area of molecular biology. The majority of studies are focusing on elucidating the impact of miRNAs in the chemoresistance of a variety of tumors, including OS. However, almost all these studies are immature (140,141). In the future, utilizing high-throughput miRNA expression analysis to identify miRNAs associated with chemoresistance should be continued. Meanwhile, further studies are required to define chemoresistance-related molecular pathways mediated by miRNA.

Following a period of silence, CSCs have returned to the study horizons again. An increase in CSC studies has revealed implications for CSCs in the drug resistance and tumor metastasis of OS. However, numerous problems remain. For instance, methods used for the isolation and identification of CSCs require a degree of improvement, and the role that CSCs play in OS metastasis and the in-depth mechanism of CSC-mediated drug resistance in OS require further systemic study (146–153).
